# The Role of Rapid Curing on the Interrelationship Between Temperature Rise, Light Transmission, and Polymerisation Kinetics of Bulk-Fill Composites

**DOI:** 10.3390/ijms26062803

**Published:** 2025-03-20

**Authors:** Danijela Marovic, Matej Par, Paulina Daničić, Ana Marošević, Gloria Bojo, Marta Alerić, Svenia Antić, Krunoslav Puljić, Ana Badovinac, Adrian C. Shortall, Zrinka Tarle

**Affiliations:** 1 University of Zagreb School of Dental Medicine, Gundulićeva 5, 10 000 Zagreb, Croatia; gbojo@sfzg.unizg.hr (G.B.); maleric@sfzg.unizg.hr (M.A.); santic@sfzg.unizg.hr (S.A.); badovinac@sfzg.unizg.hr (A.B.); tarle@sfzg.unizg.hr (Z.T.); 2 Private Dental Practice, 10 000 Zagreb, Croatia; danicic.paulina@gmail.com; 3 Private Dental Practice, 10 430 Samobor, Croatia; ana.marosevic@hotmail.com; 4 University of Zagreb Faculty of Economics & Business, Trg J. F. Kennedyja 6, 10 000 Zagreb, Croatia; kpuljic@efzg.hr; 5 School of Dentistry, The University of Birmingham, Birmingham B15 2TT, UK; a.c.c.shortall@bham.ac.uk

**Keywords:** light transmittance, degree of conversion, temperature rise, dental composites, rapid curing

## Abstract

The first seconds of light curing are crucial for the development of most properties of dental composites, especially for the 3s high-irradiance curing. This study investigated the influence of rapid high-irradiance curing on temporal development of temperature, transmittance and conversion of bulk-fill composites. Four materials were tested: Filtek One (FO), Tetric PowerFill (PFill), Tetric PowerFlow (PFlow) and SDR flow+ (SDR+) and cured with three curing units (LCU): Valo Cordles, Bluephase PowerCure and Translux Wave in 3s (3 W/cm^2^), 10s (1 W/cm^2^) and 20s (1 W/cm^2^) curing protocols. Light transmittance was measured at 2 and 4 mm, while temperature rise and polymerisation kinetics were evaluated at 4 mm depth during 5 min. Both light transmittance and temperature rise were greatest for SDR+ > PFlow > PFill > FO. The 20s curing protocol resulted in the highest degree of conversion (DC) for all materials and LCUs, but also contributed to the greatest temperature rise. Rapid curing with the 3s protocol caused the lowest temperature rise and the shortest time to reach maximum temperature. The polymerisation and temperature kinetics were strongly dependent on the material. The DC of PFill was statistically similar for 3s, 10s or 20s curing with BPC. Rapid curing is only recommended for materials developed for this purpose.

## 1. Introduction

Dental composites, as a material group, exhibit a high degree of heterogeneity [1,2,3]. Even when their composition is entirely known, the phenomena occurring at the molecular level remain insufficiently explored [1,2,4]. Moreover, the parameters evaluated under laboratory conditions are often regarded in a static context, quantified, and compared in attempts to identify the optimum material for clinical applications [5,6]. In actuality, the events following the illumination of the composite are highly dynamic, wherein light transmission, temperature increase, viscosity, crosslinking, and conversion development interact in an intricate interplay during the polymerisation [7,8,9,10,11,12,13]. The initial seconds following light illumination are critical in determining the trajectory of the development of mechanical properties, shrinkage, thermal expansion, and water absorption [8,13,14,15,16,17,18].

The quantity of photons emitted from the light-curing unit (LCU) that reach the bottom of a 2 or 4 mm thick sample is influenced not just by the light irradiance and wavelengths from the curing unit, but also significantly by the quantity of fillers, their dimensions and morphology, as well as the refractive indices of fillers and monomers, among other factors [15,19,20,21,22,23]. The photons that reach these depths excite the photoinitiators, elevating them to higher energy states and converting them into free radicals that initiate the polymerisation reaction, albeit with a certain time delay after the events on the surface [15,24].

The two-component camphorquinone/tertiary amine system remains the most commonly used photoinitiator in dental composites, capable of generating one free radical per molecule when exposed to wavelengths ranging from 360 to 510 nm [25,26]. In contrast, single-component Norrish type I photoinitiators undergo alpha-cleavage when exposed to wavelengths below 350 nm, resulting in the formation of two free radicals per molecule. This leads to a higher molar extinction coefficient and an increased quantum yield, suggesting that they are theoretically more effective as photoinitiators [25,27,28].

The factors mentioned above indicate that the formation of free radicals declines with increasing distance from the light source, as well as time, reducing the number of initiation nuclei for polymerisation [7,29]. This presents a significant challenge for bulk-fill composite materials, which are applied in layers of 4–5 mm thickness and must be adequately polymerised throughout each layer. Consequently, manufacturers have engineered bulk-fill materials to be relatively translucent to light [19]. This translucency is primarily achieved by incorporating large filler particles, which reduce light scattering on their surfaces, as well as by aligning the refractive indices of fillers and resins while minimising the use of pigments and opacifiers [30,31,32]. Furthermore, Norrish type I photoinitiators are utilised to maximise the quantum yield in some bulk-fill composites [26].

In addition to the placement in thick 4 mm layers, it is claimed that some recent bulk-fill composites can be polymerised for a significantly shorter time of only 3 s under a light irradiance of 3 W/cm^2^ [33,34]. Tetric PowerFill (Ivoclar, Schaan, Liechtenstein) achieves this by incorporating ß-allyl sulphone, a polymerisation modifier that enables reversible addition–fragmentation chain-transfer (RAFT) polymerisation [33,35]. Its flowable counterpart, Tetric PowerFlow (Ivoclar), has been engineered for 3s curing due to its very high light transmittance in the unpolymerised state [36,37,38,39]. Both materials utilise camphorquinone and germanium-based photoinitiator systems, Norrish type I and II initiators [33]. RAFT polymerisation is also utilised in the Filtek One Bulk Fill (3M ESPE, Seefeld, Germany), but the manufacturer does not recommend rapid curing [40].

RAFT polymerisation is a recent innovation in the field of dental composite resins that aims to overcome some of the disadvantages of traditional free radical polymerisation [41,42]. By incorporating RAFT agents into the resin matrix, this technique allows better control over the polymer architecture and molecular weight distribution than conventional free radical methods [42,43,44]. While free radical polymerisation remains the primary mechanism for most dental composites, RAFT modification can potentially improve properties such as depth of cure and polymerisation kinetics, enabling ultra-fast cure times of as little as 3 s for some bulk-fill materials [33,41,43,44]. The molecular processes that occur during combined RAFT and free radical polymerisation in dental composites are complex. In the initiation step, photoinitiators generate free radicals when exposed to light. These radicals then start polymerisation by reacting with monomers and initiating the chain growth process typical of radical polymerisation. In the presence of RAFT agents (such as β-allyl sulfone in Tetric PowerFill), the growing polymer chains undergo a series of addition–fragmentation steps. The propagating radical reacts with the RAFT agent and forms an intermediate RAFT adduct radical [41,44]. This adduct can fragment and either return to the original reactants or release a new initiating radical and form a dormant polymeric compound [42,44]. A RAFT equilibrium is established in which the active and dormant chains rapidly exchange providing living characteristics to the polymerisation [42]. The RAFT process provides better control over polymer architecture and molecular weight distribution than conventional free radical polymerisation [42,44]. Termination still occurs, but at a slower rate, with the active chains undergoing bi-radical termination to form the dead polymer [42].

As an exothermic chemical reaction, free radical polymerisation is accompanied by a temperature rise within the composite [45,46,47,48,49]. With a higher number of monomers being added to the polymer chains, both temperature and the modulus of the polymerising network increase [45]. This is significant because the increasing temperature reduces composite viscosity and can delay the vitrification of the polymer, also prolonging the onset of gelation and facilitating the monomer mobility [14]. This could accelerate the polymerisation rate and enhance the final degree of conversion (DC) by increasing the likelihood of collisions with the propagating chain. Moreover, additional heating from the curing unit, particularly at very high irradiances [50], can contribute to this phenomenon [51]. This interrelationship of temperature, conversion, and light transmittance regarding the 3s polymerisation of contemporary bulk-fill composite materials has not yet been investigated.

A growing variety of LCUs available today feature short, high-irradiance polymerisation modes, utilising technologies from LED to laser lights [28,50,52,53,54]. Although they may appear similar at first glance, variations in their spectral properties, chip placement, and light beam might significantly influence heat production, light transmission, and ultimately, the DC of the materials [50,55]. Par et al. have shown that even minor differences in the emission peaks of various curing units, varying by just 9 nm, can significantly influence light transmission of bulk-fill composite materials [56].

Even though rapid polymerisation is designated only for specific materials, practitioners might find themselves tempted to use this shortcut, even for other types of composite materials. Therefore, it is necessary to compare materials for rapid curing (Tetric PowerFill, Tetric PowerFlow) to other bulk-fill materials not intended for such type of polymerisation, such as Filtek One Bulk Fill and SDR flow+ Bulk Fill Flowable (Dentsply Sirona, Charlotte, North Carolina, SAD). The aim of this study was to investigate the real-time evolution of temperature, light transmittance, and conversion development of bulk-fill composites in different polymerisation modes and durations of light illumination. The null hypotheses were:

(I) There is no difference between different materials in light transmittance, temperature rise, and degree of conversion when 3s curing is used in comparison to other curing protocols.
(II) There is no difference in light transmittance, temperature rise, and degree of conversion of the same material when different curing units are used for 3s curing.

## 2. Results

### 2.1. Radiant Exitance of LCUs

The spectra of VALO Cordless showed three peaks, at 401, 448 and 460 nm; Bluephase PowerCure had two spectral peaks at 410 and 448 nm, while Translux Wave had one spectral peak (453 nm).

Table 1 shows the average values of the radiant exitance (n = 5) measured with the MARC Light Collector spectrometer at the top surface sensor at 0 mm distance and the total energy emitted during each curing protocol in the 360–540 nm range. The diameter of the active emission surface was measured for each device using a digital calliper and was 9.5 mm for Valo Cordless, 8.5 mm for Bluephase PowerCure and 7.5 mm for Translux Wave.

### 2.2. Light Transmission

The blue and violet light transmission was significantly reduced with the increasing thickness from 2 to 4 mm, for all the materials and curing units.

**Figure 1:**
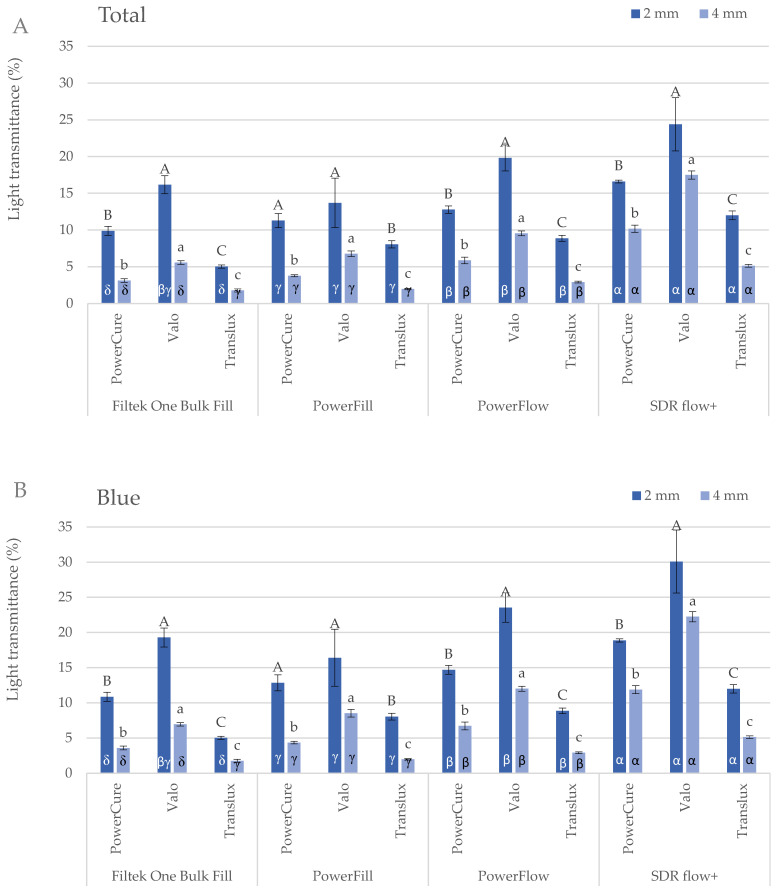

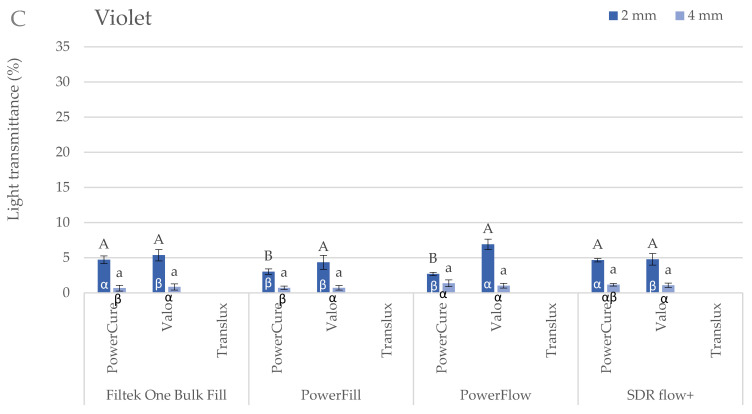
Total (**A**), blue (**B**) and violet (**C**) light transmittance (mean values ± 1 standard deviation) of the tested materials measured at 2 and 4 mm depth. Identical uppercase letters denote statistically similar groups within each material (comparisons among 3 curing units) at 2 mm depth. Identical lowercase letters denote statistically similar groups within each material (comparisons among 3 curing units) at 4 mm depth. Identical Greek letters denote statistically similar groups within a curing unit (comparisons among materials).

When **comparing the curing devices** in terms of the total and blue part of the spectrum **within each material**, Valo Cordless shows the highest light transmittance, followed by Bluephase PowerCure and Translux. For the violet part of the spectrum, only Valo Cordless and Bluephase PowerCure were compared. The transmission of violet light was 4–6 times lower than that of blue light at a thickness of 2 mm, while it was 5–20 times lower for 4 mm thick samples. The transmission of violet light at 2 mm depth was similar for both Valo Cordless and Bluephase PowerCure in Filtek One and SDR+, while Valo Cordless showed higher values in Tetric PowerFill and Tetric PowerFlow. However, for 4 mm deep samples, the transmittance of violet light was similar for all three LCUs, ranging from 0.7 to 1.4% (Figure 1).

**Figure 2:**
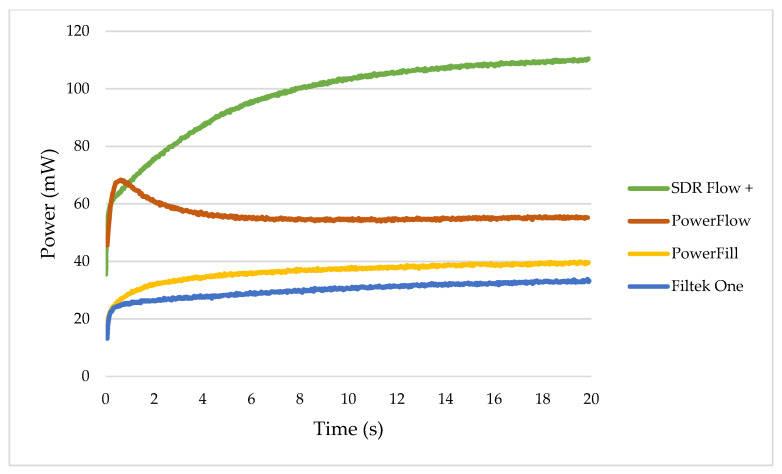
Example of the variations in transmitted power with exposure time through the 4 mm specimens of tested materials cured with Bluephase PowerCure.

Comparing **the different materials for the same curing device**, the total and blue light transmittance was highest for SDR+, followed by Tetric PowerFlow, Tetric PowerFill and Filtek One, both at 2 and 4 mm thickness (Figure 2). The violet light transmission of Bluephase PowerCure was highest for Filtek One and SDR+ at 2 mm and for PowerFlow and SDR+ at 4 mm. When illuminated with Valo Cordless, the violet light transmittance was highest for Tetric PowerFill at 2 mm and similar for all tested materials at 4 mm.

### 2.3. Temperature Rise

**Figure 3:**
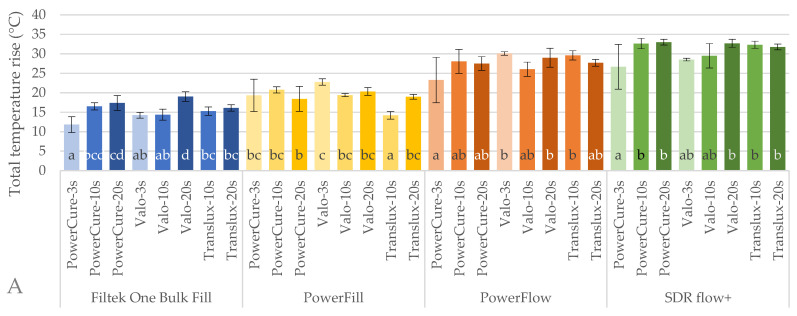

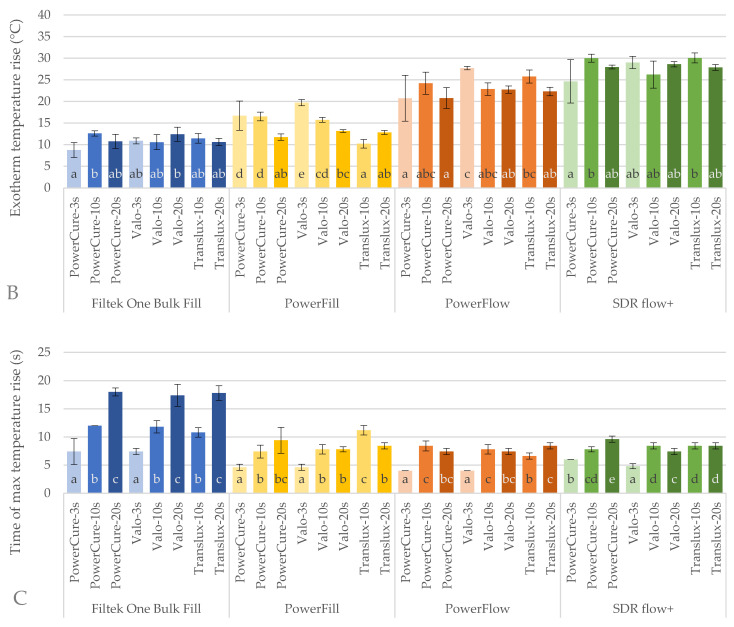
Total (**A**) and exotherm (**B**) temperature rise as well as the time to reach maximum temperature rise (**C**) (mean values ± 1 standard deviation) during 5 min post-illumination start of the tested materials measured at 4 mm depth. Identical letters denote statistically similar groups within each material.

Maximum total and exotherm temperature rise as well as the time to reach maximum temperature rise at 4 mm depth during polymerisation and 5 min after the start of illumination are depicted in Figure 3.

Total temperature rise (Figure 3A) ranged from 11.8 ± 2.0 °C for Filtek One with 3s protocol with Bluephase PowerCure to 32.6 ± 1.3 °C for SDR flow+ with 10s protocol with the same curing unit. Both total and exotherm temperature rise values were always the highest for SDR+ followed by Tetric PowerFlow, Tetric PowerFill and Filtek One with the lowest temperature rise. The exception is the 3s polymerisation protocol with Valo Cordless, which caused greater total temperature rise for Tetric PowerFlow than for SDR+. This difference was not present for the exotherm temperature rise.

When comparing different curing protocols within each material, the lowest **total**temperature rise ($T_{tot}$) was with 3s protocol of Bluephase PowerCure, except for Tetric PowerFill. For flowable materials, all the other polymerisation protocols resulted in a similar $T_{tot}$, while for sculptable, there was a material-specific behaviour. Tetric PowerFill showed a similar $T_{tot}$ for all the protocols with Bluephase PowerCure.

Similarly, in comparison of different curing protocols within each material, the lowest **exotherm** temperature rise ($T_{exo}$) was with 3s protocol of Bluephase PowerCure, except for Tetric PowerFill (Figure 3B). The maximum T_exo_ was recorded for SDR flow+ for 10s protocol with Translux curing unit, reaching 30.1 ± 1.1 °C.

Contrary to temperature rise, the **time to reach the maximum temperature** ($t_{temp}$) was the greatest for Filtek One, especially for longer curing times (Figure 3C). The 3s curing protocol, regardless of the curing unit, influenced the shortest $t_{temp}$. The time when the maximum temperature was reached for 3s protocols exceeded the illumination time.

### 2.4. Polymerisation Kinetics

**Figure 4:**
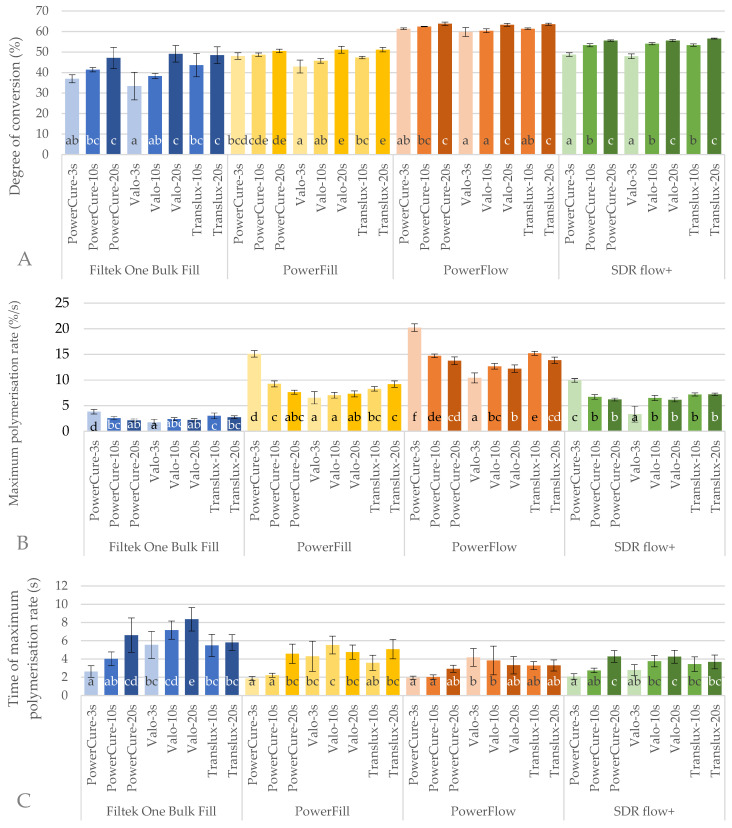
Degree of conversion attained after 5 min post-illumination (**A**), maximum reaction rate (**B**) and time to reach maximum polymerisation rate (**C**) (mean values ± 1 standard deviation) of tested materials measured at 4 mm depth. Identical letters denote statistically similar groups within each material.

The degree of conversion attained after 5 min post-illumination, maximum reaction rate and time to reach maximum reaction rate are shown in Figure 4.

Comparing the materials, Tetric PowerFlow was the material with the highest ${DC}_{5min}$ and was followed by SDR flow+, Tetric PowerFill and Filtek One, for all curing protocols (Figure 4A). There was also no difference between curing protocols regarding the maximum polymerisation rate, but the rank order of materials was changed. The material with the highest number of converted groups per second was Tetric PowerFlow, followed with Tetric PowerFill, SDR flow+, and Filtek One.

Within each material, the highest **DC_5min_** was reached with 20s protocol regardless of the curing unit used, while 3s with Valo Cordless gave the lowest ${DC}_{5min}$ for all the materials. Additionally, similarly low values were achieved with 3s-PowerCure protocol for SDR flow+. For Tetric PowerFill, there was no statistical difference between any of the curing protocols with Bluephase PowerCure.

The highest **maximum polymerisation rate** (Figure 4B) within each material was reached with 3s-PowerCure (20.2 ± 0.7%/s for PowerFlow), while the slowest rate was the result of the 3s-Valo protocol (1.7 ± 1.5%/s for Filtek One).

The shortest **time to reach the maximum polymerisation rate** (Figure 4C) was generally present for the 3s-PowerCure and 10s-PowerCure protocols. In contrast, longer curing times were generally associated with a longer time to reach the maximum polymerisation rate. The shortest time to reach the maximum polymerisation rate was recorded for Tetric PowerFill with 3s-PowerCure, which amounted to 1.9 ± 0.2 s, and the longest for Filtek One, which amounted to 8.3 ± 1.3 s.

**Figure 5:**
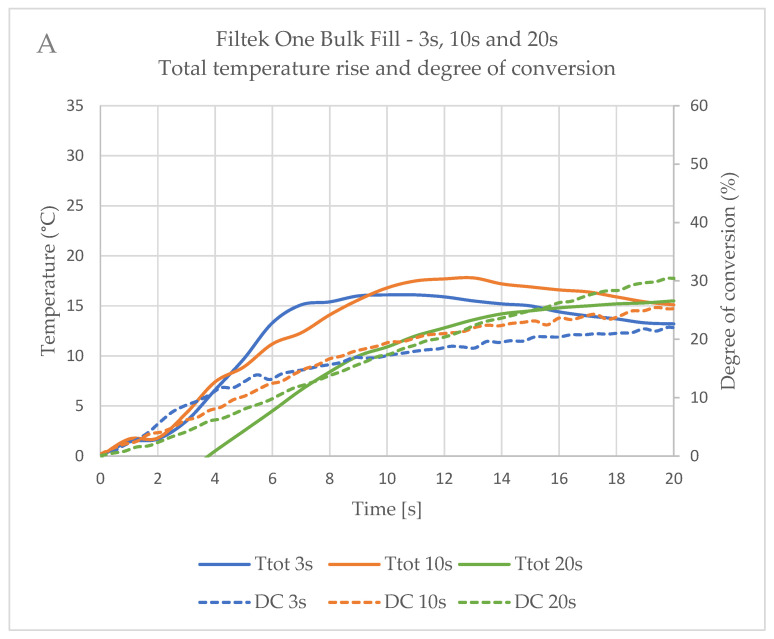

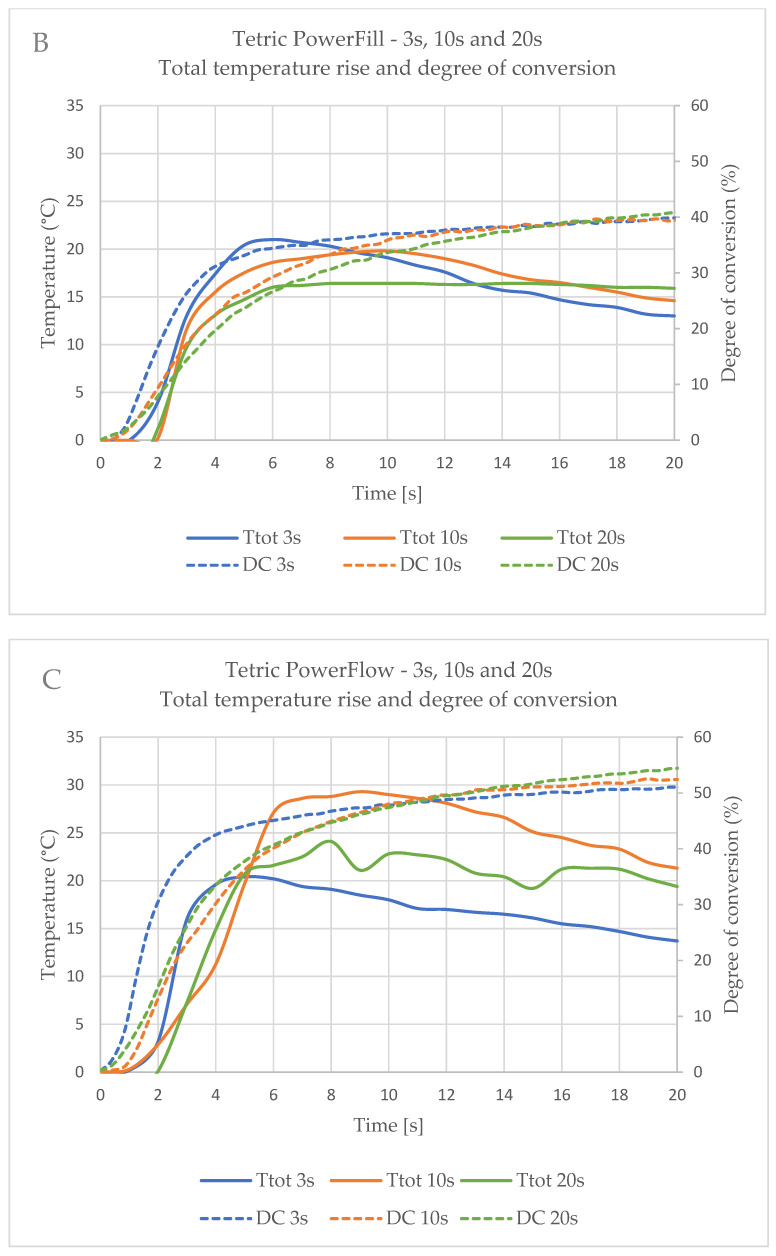

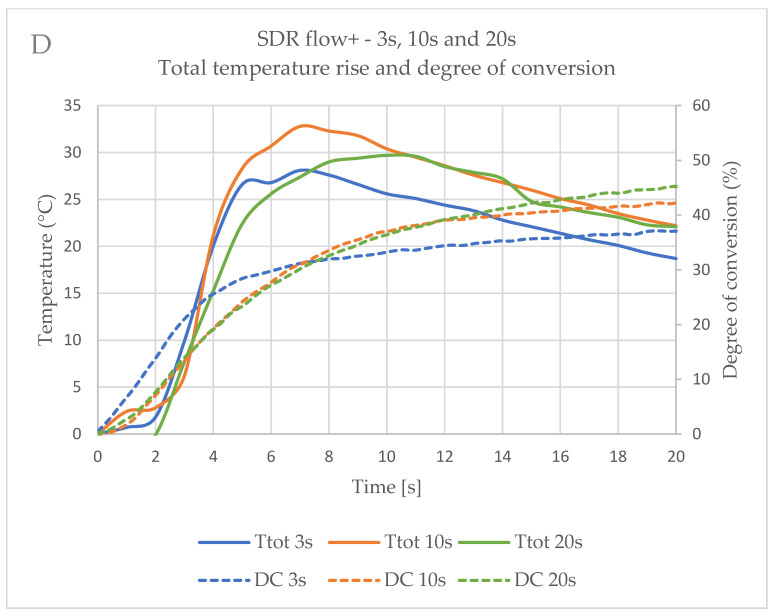
Coupled real-time thermal (total temperature rise—Ttot) and conversion (DC) data for tested materials polymerised with 3s, 10s or 20s protocol of the Bluephase PowerCure: (**A**) Filtek One Bulk Fill; (**B**) Tetric PowerFill; (**C**) Tetric PowerFlow; and (**D**) SDR flow+.

Figure 5 shows the real-time data for the DC and temperature development during the first 20 s after the start of illumination for one curing device, Bluephase PowerCure, comparing the three polymerisation protocols. For all materials tested, the 3s protocol resulted in a rapid increase in DC, but the 10s and 20s protocols achieved higher DC values in the first 20 s. The exception was Tetric PowerFill, which achieved similar values for all three protocols with this curing device. The majority of the temperature rise started up to 4 s later than the DC rise.

### 2.5. Pearson Correlation

Table 2 shows the results of Pearson correlation analysis for all the curing units and curing protocols. A moderate positive correlation was found between ${DC}_{5min}$ and the total temperature, and a somewhat lower correlation to the exotherm temperature. Light transmittance was not correlated to ${DC}_{5min}$, but there was again a moderate correlation with the total temperature, and exotherm temperature.

When analysing the correlations separately for each curing protocol, it was found that none of the parameters were correlated to the ${DC}_{5min}$. However, a strong correlation was found between total and exotherm temperature (Pearson correlation coefficient between 0.986 and 1.000, *p* < 0.001), for each curing protocol. Additionally, there was a strong correlation between light transmittance and exotherm temperature rise (Pearson correlation coefficient = 0.957, *p* = 0.043) for Bluephase PowerCure at 10s and between light transmittance and total temperature rise (Pearson correlation coefficient = 0.960, *p* = 0.040) for Bluephase PowerCure at 20s.

## 3. Discussion

This study investigated the real-time evolution of temperature, light transmittance, and DC of bulk-fill composites during and shortly after light irradiation, especially in rapid, high-irradiance light curing. The parameters varied significantly with material composition and curing protocols, so the first hypothesis was rejected. Among the tested light-curing protocols, 20s curing consistently achieved the highest DC across materials. However, shorter 3s protocols minimised temperature rise, offering potential benefits for reducing thermal risks but at the expense of polymerisation efficiency.

The results showed a significant decrease in light transmittance with increasing material thickness from 2 mm to 4 mm for all tested materials and LCUs. The results are consistent with Lambert’s law, which states that light transmission decreases with increasing material thickness due to absorption and scattering at the surface of the filler particles [19,57,58,59,60,61]. Highly filled materials such as Filtek One and Tetric PowerFill showed greater light attenuation with increasing thickness than less filled materials such as SDR+ and Tetric PowerFlow.

SDR+ had the highest total and blue light transmission, followed by Tetric PowerFlow, Tetric PowerFill and Filtek One. This trend generally correlates with the increasing filler content [11,12,19,21,62]. However, exceptions were observed in SDR+ and Tetric PowerFlow, which have a similar filler content but differ in resin compositions. SDR+ is characterised by larger filler particles (approx. 20 µm), which results in a smaller surface area for light scattering [19,20,21]. SDR+ is also known for its high light transmittance [11,19,63], which constantly increases with polymerisation time. The other three materials in this study are characterised by the increasing refractive index mismatch between the fillers [33,40]. It is noteworthy that the opacity of Tetric PowerFlow increased quickly after the initiation of polymerisation, characterised by a short (0.6 s) increase in light transmittance, followed by its decline to the end of the measurement (Figure 2). This trend probably explains why Tetric PowerFlow has a lower overall and blue light transmission than SDR+ despite a lower filler volume.

The total and blue light transmittance values were similar across all materials and LCUs tested. This is expected because blue light dominates the emission spectrum of every LCU used in this study. Blue light penetrated deeper than violet light, with violet light transmittance dropping to less than 1.5% at the bottom of the 4 mm samples. In substances with high light scattering like composite resins, higher-energy ultraviolet wavelengths fail to penetrate deeply [21,63,64]. In contrast, lower-energy blue wavelengths can more effectively penetrate a 4 mm thick composite sample [56,57,65]. Similar findings were found in our previous studies [56,65].

In this study, the temperature rise at 4 mm depth was evaluated by two different measurements: the total temperature rise and exotherm temperature rise. The total temperature rise reflects the combined effects of the exothermic polymerisation reaction and the external heat from the LCU [66,67]. The total temperature rise represents the actual temperature increase that can contribute to increased translucency and lower viscosity of the resin [45]. On the other hand, the exotherm temperature rise approximates the heating contribution of the polymerisation reaction alone [68].

Both types of temperature measurements positively correlated with light transmittance, similar to Lee and Lee [49]. Less filled materials showed higher light transmittance and a higher temperature rise. The same order of materials was observed: SDR+ showing the highest temperature increase, followed by Tetric PowerFlow, Tetric PowerFill and Filtek One. This correlation is related to the filler volume [20,69], as highly filled materials contain less resin and hence a lower number of reactive C=C groups, leading to a lower temperature rise. This conclusion is consistent with the results of another study that found a lower temperature rise in highly filled experimental composites compared to unfilled or low-filled composites [45].

Temperature rise is usually studied in relation to its potential to induce unwanted inflammatory pulpal response [46,48,70]. However, this was not the focus of the current study. Instead, the intention was to explore if the high radiant exitance in the 3s curing protocol generates increased temperature and, consequently, an increase in DC. In the present study, a positive correlation was found between total and exotherm temperature and the $DC_{5min}$; however, not for 3s curing.

Higher temperatures can also accelerate the polymerisation reaction, leading to improved conversion rates [45,47]. They reduce the viscosity of the resin, increase the kinetic energy of the reactive monomers and enhance the probability of collisions between reactive sites, thereby improving the DC [14].

Howard et al. noted that the maximum temperature rise coincides with the peak reaction rate during the early phase of polymerisation, measured in the middle of a 0.8 mm thick specimen [45]. This thermal boost supposedly accelerates the conversion process, especially in the gel phase of the reaction, where molecular mobility is still relatively high [45]. However, in the present study, the time to reach maximum temperature was 2–3 times longer than the time to reach maximum reaction rate. This time delay in reaching the maximum temperature could be explained by the differences in the thickness of the specimen or even measurement techniques, i.e., thermocouple in the study by Howard et al. vs. thermal camera in our study. The contact measurement using a thermocouple records the temperature locally within the specimen, whereas the non-contact measurement using a thermal camera results in slower temperature readings. This delay is likely due to the time required for heat conduction through the specimen to its surface, where the measurement is made by the thermal camera. Furthermore, the time required for heat conduction through a 4 mm thick specimen in the present study is probably higher than for a 0.8 mm thick specimen. However, this needs to be investigated in further studies.

Valo’s 3s protocol was the only protocol that showed similar times for reaching maximum temperature and maximum reaction rate. However, it did not lead to a DC increase. On the contrary, the 3s protocol with Valo Cordless consistently produced the lowest DC, although Valo Cordless consistently produced the highest total and blue transmittance. However, the total energy emitted by Valo’s 3s protocol (7.7 J/cm^2^) was more than 15% lower than that of Bluephase PowerCure’s 3s protocol (9.1 J/cm^2^), which probably influenced the low DC value.

An ideal polymerisation protocol would achieve a high DC, while maintaining slow polymerisation rate with a delayed peak in the polymerisation rate to minimise the consequences of the polymerisation shrinkage stress. This protocol should also minimise temperature rise with a significant delay in reaching the maximum temperature to avoid overheating the pulp. At the same time, the restorative procedure should be quick, allowing the placement in thick layers that are insensitive to the operator’s mistakes. The composite material should have sufficient opacity to disguise the unwanted discolorations of the tooth cavity. However, this ideal situation is difficult to achieve.

The most important outcome of any polymerisation protocol is the DC, the fundamental property that influences most other properties, from mechanical to biological [1,12,71,72]. In this study, light irradiation with the 20s protocol consistently yielded the highest DC for all materials tested, regardless of the LCU used. However, it should be noted that the energy received by the samples, known as the radiant exposure (the product of time and irradiance), was at least twice as high with the 20s protocol compared to the samples with the 3s and 10s protocols (Table 1). At the same time, the additional advantage of the 20s protocol was that the polymerisation rate, temperature rise and time to reach the maximum temperature and polymerisation rate were not increased, but were similar to the 10s protocol for most LCU/material combinations. On the other hand, the heat generation by the LCU in the 20s protocol was about twice as high as in the 3s or 10s protocol. Similar results were observed in the study by Thanoon et al. [73]. It appears that the higher total energy delivered to the samples in the 20s protocol had a greater effect on the temperature rise than the higher irradiance in the 3s protocol.

The DC values achieved with the 3s and 10s protocols for each LCU showed no significant difference for any of the materials except SDR+. Both the 3s and 10s protocols delivered similar energy. The advantage of the 3s protocol, apart from the time saved in clinical work, is the reduction in temperature rise. The 3s protocol generally showed the lowest temperature rise despite the highest radiant exitance. One exception was Tetric PowerFill, which showed a higher temperature rise in the 3s protocol than the other materials. This is probably due to the material design, which contains ß-allyl sulphone as a RAFT agent and promotes the rapid formation of short polymer chains in the step-wise polymerisation reaction concurrently with conventional radical polymerisation [44]. Along with highly efficient initiation of the polymerisation reaction by germanium-based photoinitiator Ivocerin, this led to exothermic temperature rise. Elevated temperatures in the combined RAFT and radical polymerisation lead to accelerated reaction rates, faster radical formation and increased monomer-radical collisions, resulting in faster polymerisation. These changes can influence the molecular weight distribution of the polymer and affect the overall mechanical properties of the dental composite [74].

The second hypothesis, that there is no difference between 3s protocols from different LCUs, is also rejected. The 3s protocol of Valo Cordless generally produced a lower polymerisation rate with a longer time to reach the maximum polymerisation rate, despite the higher light transmittance than Bluephase PowerCure. On the other hand, the total and exotherm temperature as well as the time to reach the maximum temperature were almost always the same for both LCUs in the 3s protocol. The only materials that deviated from this were Tetric PowerFill and PowerFlow, which reached higher temperatures with 3s Valo Cordless than with 3s Bluephase PowerCure, consistent to findings of Odum et al. [75]. However, the ${DC}_{5min}$ was only lower with Tetric PowerFill for 3s Valo. The differences were most likely related to the different irradiances of the two LCUs. All LCUs in the present study have been used regularly for approximately one year prior to this study, were fully charged and showed no visible damage to the light guide tip. The manufacturers of both Valo Cordless and Bluephase PowerCure recommend a curing time of 3s in their Xtra Power and 3s programmes [33,76]. Valo Cordless had lower radiant exitance (2522.6 mW/cm^2^) than specified by the manufacturer, which was significantly lower than that of Bluephase (3045.6 mW/cm^2^).

The material viscosity had the greatest influence on the results of the current study. In particular, the low-filled materials, SDR+ and Tetric PowerFlow, behaved similarly and there were some similarities between the two high-filled materials, Filtek One and Tetric PowerFill.

Both low-filled materials showed the highest light transmittance and the highest temperature rise, a short time to reach the maximum temperature and the highest DC. While SDR+ dominated in light transmittance and temperature rise, it did not contribute to achieving the highest DC. Instead, the best polymerised material was Tetric PowerFlow, which also showed the fastest reaction rate. This quick polymerisation reaction could be due to the presence of two types of photoinitiators, in contrast to SDR, which only contained camphorquinone. Although only 1.4% of the incident violet wavelengths reached the 4 mm depth of Tetric PowerFlow, this was apparently sufficient to activate the Ivocerin. In addition, Ivocerin has a broad absorption spectrum and it is possible to activate it with blue wavelengths. This resulted in the highest maximum reaction rate and the best DC in this study of 63.7 ± 0.8% for the 20s cure with Bluephase PowerCure. However, 3s curing resulted in comparable values of 61.3 ± 0.4%, albeit statistically different.

On the other hand, high-filled materials showed lower light transmittance and a lower temperature rise as well as a longer time to reach the maximum temperature rise, consistent with their filler volume. Tetric PowerFill showed higher values than Filtek One. The manufacturers of both materials state that the RAFT agents are present, but Filtek One also contains AUDMA, a high molecular weight monomer, while Tetric PowerFill is claimed to form uniform short chains. The monomer composition of Filtek One appeared to affect the reaction rate, as long AUDMA molecules slowed down the polymerisation reaction. Together with the highest filler volume among all materials tested in this study, this resulted in the slowest reaction rate and the longest time to reach the maximum for Filtek One. The 3s protocol with Valo Cordless was particularly unfavourable for the DC of this material, resulting in only 33.4 ± 6.7%. 3s curing with Valo Cordless also resulted in a significantly lower DC for Tetric PowerFill (42.9 ± 3.2%). On the other hand, when cured with Bluephase PowerCure, the high mobility of the short chains of Tetric PowerFill contributed to the second fastest reaction time and statistically similar DC values for polymerisation with the 3s, 10s or 20s protocols of (48.0 ± 1.6% for 3s and 50.5 ± 0.9% for 20s).

It should be noted that all tests were carried out at a depth of 4 mm to simulate the most challenging conditions occurring at the bottom of a layer with the maximum allowable thickness. Measures were taken to ensure uniformity of conditions for all tests. However, this study has several limitations, including the in vitro conditions and the focus on a specific group of bulk-fill materials. It does not reflect the real clinical situation, where in some cases, when working in hard-to-reach areas or when the mouth opening is restricted, 3s curing could jeopardise the clinical outcome. Future research should also investigate the long-term clinical performance of bulk-fill composites under rapid curing protocols.

## 4. Materials and Methods

In this study, four bulk-fill materials were tested, two flowable (Tetric PowerFlow and SDR flow+ Bulk Fill Flowable) and two sculptable (Tetric PowerFill and Filtek One Bulk Fill), as shown in Table 3.

This study utilised three LCUs that had been in clinical use for one year, following these light-curing protocols with nominal irradiance values:

(I) VALO Cordless (Ultradent, South Jordan, UT, USA; VC) as LCU with three spectral peaks:
  - 3s protocol: 3 s with 3 W/cm^2^,
  - 10s protocol: 10 s with 1 W/cm^2^, and
  - 20s protocol: 20 s with 1 W/cm^2^.
(II) Bluephase PowerCure (Ivoclar; BPC) As LCU with two spectral peaks:
  - 3s protocol: 3 s with 3 W/cm^2^,
  - 10s protocol: 10 s with 1 W/cm^2^, and
  - 20s protocol: 20 s with 1 W/cm^2^.
(III) Translux Wave (Kulzer GmbH, Hanau, Germany; TW) as LCU with one spectral peak:
  - 10s protocol: 10 s with 1 W/cm^2^, and
  - 20s protocol: 20 s with 1 W/cm^2^.

### 4.1. Characterisation of the Curing Units

The LCUs were characterised using a National Institute of Standards and Technology (NIST)-referenced and calibrated spectrometer MARC Light Collector (BlueLight Analytics Inc., Halifax, NS, Canada). Radiant exitance, emission spectrum and total energy of the LCUs were measured on the 16 mm diameter collection port (top surface sensor) with five repetitions per curing protocol.

### 4.2. Light Transmittance

Light transmittance was measured in real time and at room temperature through the bulk-fill composite specimens. Cylindrical Delrin moulds (h = 2 mm and 4 mm, internal d = 6 mm) were filled with uncured material. The top and bottom openings of the mould were covered with polyethylene terephthalate (PET) film and pressed with a glass slide until the mould was in contact to remove the excess material. The Delrin^®^ mould with the uncured resin and PET sheets on both sides was placed on the bottom surface sensor (aperture diameter 4 mm) of the NIST-calibrated spectrometer MARC Light Collector with the flush side down [57]. The materials (n = 5 samples per material/thickness/curing unit) were polymerised with each curing unit for 20 s with the nominal value of ≈ 1 W/cm^2^.

The tip of the LCU was perpendicular and in direct contact with the top PET film on the uncured material. The position of the LCUs was controlled and fixed using the MARC Accessory bench (BlueLight Analytics Inc.). The data were recorded with the MARCLC 5.0 software (BlueLight Analytics Inc.). The real-time irradiance at the bottom of the specimen at a distance of 2 mm and 4 mm was measured by the MARC Light Collector throughout light curing.

The total irradiance of the entire spectrum, the irradiance with wavelengths of 360–420 nm for the violet spectrum and 420–540 nm for the blue spectrum of Bluephase PowerCure were analysed separately. Additionally, the blue light spectrum for Valo Cordless was divided into “Blue I” from 424 to 453 nm and “Blue II” from 453 to 540 nm. For Translux Wave, the total and blue irradiance were identical and ranged from 360 to 540 nm.

### 4.3. Temperature Rise

All three curing devices with eight curing protocols were used to measure the temperature rise. Five samples per material and curing protocol were prepared, a total of 160 specimens and 320 measurements.

The uncured composite material was filled into a Teflon mould with an aperture diameter of 5 mm and a thickness of 4 mm and covered with PET film on top and bottom. The distance between the samples and the thermal camera was 9 cm.

The temperature rise during polymerisation was measured in real time on the underside of the sample (h = 4 mm, d = 6 mm) illuminated from the opposite side, using a thermal camera (ETS 320 electronics test bench camera, FLIR ExaminIR, Teledyne, Wilsonville, OR, USA) [67]. The room temperature ($T_{room}$) and the maximum temperature reached (${T1}_{max}$) after the first illumination as well as the time until the maximum temperature was reached ($t_{temp}$) were recorded. After cooling to room temperature, the samples were re-illuminated with the lamp and the second maximum temperature reached (${T2}_{max}$) was recorded to distinguish the **exotherm temperature rise** (${{T_{exo}=T1}_{max}-T2}_{max}$) due to the polymerisation reaction from the **total temperature rise** (${T_{tot}=T1}_{max}-T_{room}$) due to the influence of the polymerisation device and the exotherm temperature.

### 4.4. Polymerisation Kinetics

The polymerisation kinetics was evaluated using a Fourier transform infrared (FTIR) spectrometer (Nicolet iS50, Thermo Fisher, Madison, WI, USA) with an attenuated total reflectance (ATR) accessory at room temperature.

The uncured composites (n = 5) were placed in custom-made silicone moulds (d = 3 mm, h = 4 mm), covering the ATR diamond and a PET foil on each specimen’s top surface, using the aforementioned light-curing units in designated light-curing protocols. Light curing was activated and FTIR spectra were recorded in real time at a rate of 2 spectra/s for 5 min, with 4 scans and a resolution of 8 cm^−1^ [77]. We tested five specimens per material and light-curing protocol (n = 5).

The changes in the ratios of absorbance intensities of the aliphatic band at 1638 cm^−1^ and the reference band were used to calculate the DC (in %):

(1) $$DC=\left(1-\frac{{\left(\frac{absorbance\left(1638\;{cm}^{-1}\right)}{absorbance\left(reference\right)}\right)}_{cured}}{{\left(\frac{absorbance\left(1638\;{cm}^{-1}\right)}{absorbance\left(reference\right)}\right)}_{uncured}}\right)\cdot 100$$

The spectral band at 1608 cm^−1^ was used as a reference for all composites except for Filtek One Bulk Fill, for which an alternative band at 1600 cm^−1^ (C-H stretching) was used as a reference [78].

The DC data were plotted as a function of time and calculated the first derivatives to represent the rate of polymerisation. The obtained polymerisation rate was plotted as a function of time to determine the maximum polymerisation rate ($R_{max}$) and the time required to reach the maximum polymerisation rate ($t_{maxDC}$). Additionally, the DC values reached at the end of the 5-min observation period (${DC}_{5min}$) were calculated.

### 4.5. Statistical Analysis

The Shapiro–Wilk test and normal Q–Q plots showed that there were no significant deviations from the normal distribution. The light transmission was compared using a three-way ANOVA with the factors “thickness”, “curing unit”, and “material”. After significant interactions between the factors were found, one-way ANOVAs were performed to determine the effects of each factor at fixed levels of the other two factors. For temperature rise (total and exotherm) and time of maximum temperature rise, a one-way ANOVA was used to compare the combinations of curing unit and curing protocol (the combination being regarded as a single factor) within each material. In the same way, degree of conversion, maximum polymerisation rate, and time of maximum polymerisation rate were compared among the combinations of curing unit and curing protocol within each material separately. Tukey’s post hoc adjustment was used for multiple comparisons. The overall significance level was 0.05. Statistical analysis was performed using SPSS, version 26.1 (IBM, Armonk, NY, USA).

## 5. Conclusions

- The interplay between temperature rise, light transmission and polymerisation kinetics is complex and material dependent.
- Rapid curing offers several advantages, including shorter treatment time and greater patient comfort. However, the potentially lower degree of conversion in the deepest layers poses a risk to the long-term success of some bulk-fill composite restorations.
- Extended curing times with moderate irradiance (≈1 W/cm^2^) were beneficial for all tested materials.
- Rapid curing with ≈3 W/cm^2^ should be reserved exclusively for materials specifically designed for this curing protocol and is not recommended for use with other materials.

## Figures and Tables

**Table 1:** Radiant characteristics of the curing units used in this study.

Light-Curing Unit	Curing Protocol	Mean Irradiance ± s.d. at 0 mm (mW/cm^2^)	Total Energy ± s.d. from 360 to 540 nm (J/cm^2^)
VALO Cordless	3s	2522.6 ± 10.9	7.7 ± 0.0
	10s	1050.8 ± 1.8	10.6 ± 0.0
	20s	1057.0 ± 4.9	21.2 ± 0.1
Bluephase PowerCure	3s	3045.6 ± 16.3	9.1 ± 0.0
	10s	1187.4 ± 3.4	11.9 ± 0.0
	20s	1203.8 ± 4.0	24.1 ± 0.1
Translux Wave	10s	805.2 ± 3.4	7.9 ± 0.0
	20s	789.0 ± 7.1	16.1 ± 0.3

**Table 2:** The results of Pearson correlation analysis for all the curing units and curing protocols.

	DC	Total Temperature Rise	Exotherm Temperature Rise	Light Transmittance
DC	-	R = 0.742 *p* < 0.001	R = 0.641 *p* < 0.001	R = 0.217 *p* = 0.232
Total temperature rise	-	-	R = 0.973 *p* < 0.001	R = 0.609 *p* < 0.001
Exotherm temperature rise	-	-	-	R = 0.655 *p* < 0.001

**Table 3:** The composition of tested materials given by the manufacturers.

Material	LOT No.	Composition	Filler Load (wt%/vol%)
Filtek One Bulk Fill (3M ESPE) A2	NC09993	AUDMA, AFM, DDDMA, UDMA, ytterbium trifluoride, 4 to 11 nm zirconia filler, an aggregated zirconia/silica cluster filler (comprised of 20 nm silica and 4 to 11 nm zirconia particles)	≈76.5/≈58.5
Tetric PowerFill (Ivoclar) IVA	Z01SDW	Bis-GMA, Bis-EMA, UDMA, Bis-PMA, DCP, D3MA, β-allyl sulfone, barium glass, ytterbium trifluoride, copolymer, mixed oxide camphorquinone, tertiary amines, Ivocerin	76–77/53–54
Tetric PowerFlow (Ivoclar) IVA	Z03236	Bis-GMA, Bis-EMA, UDMA, DCP, barium glass, ytterbium trifluoride, copolymer, mixed oxide camphorquinone, tertiary amines, Ivocerin	68.2/46.4
SDR flow+ Bulk Fill Flowable (Dentsply Sirona) A2	2101000559	modified UDMA, TEGDMA, dimethacrylate, trimethacrylate resins, camphorquinone; ethyl-4(dimethylamino)benzoate photoaccelerator; BHT; barium-alumino-fluoro-borosilicate glass; silanated strontium alumino-fluoro-silicate glass; surface treated fume silicas; ytterbium fluoride; synthetic inorganic iron oxide pigments, and titanium dioxide	70.5/47.4

Bis-GMA: bisphenol A-diglycidyl dimethacrylate; Bis-EMA: ethoxylated bisphenol A dimethacrylate; DCP: tricyclodecane–dimethanol dimethacrylate; UDMA: urethane dimethacrylate; AUDMA: aromatic dimethacrylate; DMA: dimethacrylate; AFM: addition fragmentation monomers; TEGDMA: triethylene glycol dimethacrylate; PEGDMA: polyethylene glycol dimethacrylate; Bis-PMA: propoxylated bisphenol A dimethacrylate; D3MA: decandiol dimethacrylate; DDDMA: 1,12-dodecanediol dimethacrylate; BHT: butylated hydroxy toluene.

## Data Availability

The datasets generated during the current study are available from the corresponding author on reasonable request.

## Abbreviations

The following abbreviations are used in this manuscript:

FO	Filtek One
PFill	Tetric PowerFill
PFlow	Tetric PowerFlow
SDR+	SDR flow+
VC	Valo Cordless
BPC	Bluephase PowerCure
TW	Translux Wave
DC	Degree of conversion
LCU	Light-curing unit
RAFT	Reversible addition fragmentation chain transfer
Bis-GMA	Bisphenol A-diglycidyl dimethacrylate
Bis-EMA	Ethoxylated bisphenol A dimethacrylate
Bis-PMA	Propoxylated bisphenol A dimethacrylate
DCP	Tricyclodecane–dimethanol dimethacrylate
DMA	Dimethacrylate
UDMA	Urethane dimethacrylate
AUDMA	Aromatic dimethacrylate
TEGDMA	Triethylene glycol dimethacrylate
PEGDMA	Polyethylene glycol dimethacrylate
D3MA	Decandiol dimethacrylate
DDDMA	1,12-Dodecanediol dimethacrylate
AFM	Addition fragmentation monomers
NIST	National Institute of Standards and Technology
PET	Polyethylene terephthalate
FTIR	Fourier transform infrared
ATR	Attenuated total reflectance

## References

[1] Ferracane J.L. (2011). Resin composite-state of the art. Dent. Mater..

[2] Ilie N., Hickel R. (2009). Investigations on mechanical behaviour of dental composites. Clin. Oral. Investig..

[3] Ilie N., Hickel R. (2009). Macro-, micro- and nano-mechanical investigations on silorane and methacrylate-based composites. Dent. Mater..

[4] Haugen H.J., Ma Q., Linskens S., Par M., Mandic V.N., Mensikova E., Nogueira L.P., Taubock T.T., Attin T., Gubler A. (2024). 3D micro-CT and O-PTIR spectroscopy bring new understanding of the influence of filler content in dental resin composites. Dent. Mater..

[5] Marovic D., Haugen H.J., Par M., Linskens S., Mensikova E., Negovetic Mandic V., Leeuwenburgh S., Nogueira L.P., Vallittu P.K., Ma Q. (2024). Emerging technologies for the evaluation of spatio-temporal polymerisation changes in flowable vs. sculptable dental resin-based composites. Dent. Mater..

[6] Yadav R., Saini S., Sonwal S., Meena A., Huh Y.S., Brambilla E., Ionescu A.C. (2024). Optimization and ranking of dental restorative composites by ENTROPY-VIKOR and VIKOR-MATLAB. Polym. Adv. Technol..

[7] Watts D.C. (2023). Light-curing dental resin-based composites: How it works and how you can make it work. Front. Dent. Med..

[8] Watts D.C. (2005). Reaction kinetics and mechanics in photo-polymerised networks. Dent. Mater..

[9] Soh M.S., Yap A.U. (2004). Influence of curing modes on crosslink density in polymer structures. J. Dent..

[10] da Silva E.M., Poskus L.T., Guimaraes J.G., de Araujo Lima Barcellos A., Fellows C.E. (2008). Influence of light polymerization modes on degree of conversion and crosslink density of dental composites. J. Mater. Sci. Mater. Med..

[11] Fronza B.M., Ayres A., Pacheco R.R., Rueggeberg F.A., Dias C., Giannini M. (2017). Characterization of Inorganic Filler Content, Mechanical Properties, and Light Transmission of Bulk-fill Resin Composites. Oper. Dent..

[12] Fidalgo-Pereira R., Carpio D., Torres O., Carvalho O., Silva F., Henriques B., Ozcan M., Souza J.C.M. (2022). The influence of inorganic fillers on the light transmission through resin-matrix composites during the light-curing procedure: An integrative review. Clin. Oral. Investig..

[13] Ferracane J.L., Berge H.X., Condon J.R. (1998). In vitro aging of dental composites in water-effect of degree of conversion, filler volume, and filler/matrix coupling. J. Biomed. Mater. Res..

[14] Stansbury J.W. (2012). Dimethacrylate network formation and polymer property evolution as determined by the selection of monomers and curing conditions. Dent. Mater..

[15] Sullivan B., Kalliecharan D., Kostylev I., Earle G., Stansbury J.W., Price R.B., Labrie D. (2021). Photo-polymerization kinetics of a dental resin at a high temporal resolution. J. Mech. Behav. Biomed. Mater..

[16] Alrahlah A., Silikas N., Watts D.C. (2014). Post-cure depth of cure of bulk fill dental resin-composites. Dent. Mater..

[17] Al-Ahdal K., Ilie N., Silikas N., Watts D.C. (2015). Polymerization kinetics and impact of post polymerization on the Degree of Conversion of bulk-fill resin-composite at clinically relevant depth. Dent. Mater..

[18] Sideridou I., Tserki V., Papanastasiou G. (2003). Study of water sorption, solubility and modulus of elasticity of light-cured dimethacrylate-based dental resins. Biomaterials.

[19] Bucuta S., Ilie N. (2014). Light transmittance and micro-mechanical properties of bulk fill vs. conventional resin based composites. Clin. Oral. Investig..

[20] Ilie N., Hogg C. (2024). Kinetic of Light Transmission during Setting and Aging of Modern Flowable Bulk-Fill Composites. Materials.

[21] Arikawa H., Kanie T., Fujii K., Takahashi H., Ban S. (2007). Effect of filler properties in composite resins on light transmittance characteristics and color. Dent. Mater. J..

[22] Shortall A.C., Palin W.M., Burtscher P. (2008). Refractive index mismatch and monomer reactivity influence composite curing depth. J. Dent. Res..

[23] Shimokawa C., Sullivan B., Turbino M.L., Soares C.J., Price R.B. (2017). Influence of Emission Spectrum and Irradiance on Light Curing of Resin-Based Composites. Oper. Dent..

[24] Andrzejewska E. (2001). Photopolymerization kinetics of multifunctional monomers. Prog. Polym. Sci..

[25] Hadis M.A., Shortall A.C., Palin W.M. (2024). The power of light—From dental materials processing to diagnostics and therapeutics. Biomater. Investig. Dent..

[26] Kowalska A., Sokolowski J., Bociong K. (2021). The Photoinitiators Used in Resin Based Dental Composite-A Review and Future Perspectives. Polymers.

[27] Moszner N., Fischer U.K., Ganster B., Liska R., Rheinberger V. (2008). Benzoyl germanium derivatives as novel visible light photoinitiators for dental materials. Dent. Mater..

[28] Braga S.S.L., Price R.B., Juckes S.M., Sullivan B., Soares C.J. (2024). Effect of the violet light from polywave light-polymerizing units on two resin cements that use different photoinitiators. J. Prosthet. Dent..

[29] Labrie D., Price R.B., Sullivan B., Salazar A.M., Gautam D., Stansbury J.W., Ferracane J.L. (2022). Effect of thickness on the degree of conversion of two bulk-fill and one conventional posterior resin-based composites at high irradiance and high temporal resolution. J. Mech. Behav. Biomed. Mater..

[30] Ilie N. (2022). Resin-Based Bulk-Fill Composites: Tried and Tested, New Trends, and Evaluation Compared to Human Dentin. Materials.

[31] Clewell D.H. (1941). Scattering of Light by Pigment Particles. J. Opt. Soc. Am..

[32] Ismail E.H., Paravina R.D. (2022). Color adjustment potential of resin composites: Optical illusion or physical reality, a comprehensive overview. J. Esthet. Restor. Dent..

[33] Todd J. (2019). Scientific documentation 3s PowerCure.

[34] Marovic D., Par M., Crnadak A., Sekelja A., Negovetic Mandic V., Gamulin O., Rakic M., Tarle Z. (2021). Rapid 3 s Curing: What Happens in Deep Layers of New Bulk-Fill Composites?. Materials.

[35] Graf N., Ilie N. (2020). Long-Term Stability of a RAFT-Modified Bulk-Fill Resin-Composite under Clinically Relevant Versus ISO-Curing Conditions. Materials.

[36] Marovic D., Par M., Macan M., Klaric N., Plazonic I., Tarle Z. (2022). Aging-Dependent Changes in Mechanical Properties of the New Generation of Bulk-Fill Composites. Materials.

[37] Macan M., Marošević A., Špiljak B., Šimunović L., Par M., Marovic D., Juric-Kacunic D., Tarle Z. (2023). Proposition of New Testing Procedure for the Mechanical Properties of Bulk-Fill Materials. Materials.

[38] Klaric E., Bosnic J.V., Par M., Tarle Z., Marovic D. (2024). One-Year Evaluation of High-Power Rapid Curing on Dentin Bond Strength. Materials.

[39] Hayashi J., Tagami J., Chan D., Sadr A. (2020). New bulk-fill composite system with high irradiance light polymerization: Integrity and degree of conversion. Dent. Mater..

[40] 3M Filtek One Bulk Fill Restaurative. Shrinkage, Stress and Bulk Fill Restoratives 2020. https://multimedia.3m.com/mws/media/1317665O/1317663m-filtek-one-bulk-fill-restorative-shrink-stressand-bulk-fill-restoratives.pdf.

[41] Ilie N., Watts D.C. (2020). Outcomes of ultra-fast (3 s) photo-cure in a RAFT-modified resin-composite. Dent. Mater..

[42] Moad C.L., Moad G. (2021). Fundamentals of reversible addition–fragmentation chain transfer (RAFT). Chem. Teach. Int..

[43] Gorsche C., Griesser M., Gescheidt G., Moszner N., Liska R. (2014). β-Allyl Sulfones as Addition–Fragmentation Chain Transfer Reagents: A Tool for Adjusting Thermal and Mechanical Properties of Dimethacrylate Networks. Macromolecules.

[44] Gorsche C., Koch T., Moszner N., Liska R. (2015). Exploring the benefits of β-allyl sulfones for more homogeneous dimethacrylate photopolymer networks. Polym. Chem..

[45] Howard B., Wilson N.D., Newman S.M., Pfeifer C.S., Stansbury J.W. (2010). Relationships between conversion, temperature and optical properties during composite photopolymerization. Acta Biomater..

[46] Randolph L.D., Palin W.M., Watts D.C., Genet M., Devaux J., Leloup G., Leprince J.G. (2014). The effect of ultra-fast photopolymerisation of experimental composites on shrinkage stress, network formation and pulpal temperature rise. Dent. Mater..

[47] Trujillo M., Newman S.M., Stansbury J.W. (2004). Use of near-IR to monitor the influence of external heating on dental composite photopolymerization. Dent. Mater..

[48] Kim R.J., Son S.A., Hwang J.Y., Lee I.B., Seo D.G. (2015). Comparison of photopolymerization temperature increases in internal and external positions of composite and tooth cavities in real time: Incremental fillings of microhybrid composite vs. bulk filling of bulk fill composite. J. Dent..

[49] Lee C.H., Lee I.B. (2023). Effect of translucency and absorbance of composite on temperature change during photopolymerization. Dent. Mater. J..

[50] Price R.B., Ferracane J.L., Shortall A.C. (2015). Light-Curing Units: A Review of What We Need to Know. J. Dent. Res..

[51] Price R.B., Whalen J.M., Price T.B., Felix C.M., Fahey J. (2011). The effect of specimen temperature on the polymerization of a resin-composite. Dent. Mater..

[52] Negovetic Mandic V., Par M., Marovic D., Rakić M., Tarle Z., Klarić Sever E. (2023). Blue Laser for Polymerization of Bulk-Fill Composites: Influence on Polymerization Kinetics. Nanomaterials.

[53] Almeida R., Manarte-Monteiro P., Domingues J., Falcao C., Herrero-Climent M., Rios-Carrasco B., Lemos B.F. (2021). High-Power LED Units Currently Available for Dental Resin-Based Materials-A Review. Polymers.

[54] Rueggeberg F.A., Giannini M., Arrais C.A.G., Price R.B.T. (2017). Light curing in dentistry and clinical implications: A literature review. Braz. Oral. Res..

[55] Ilie N., Schmalz G., Fujioka-Kobayashi M., Lussi A., Price R.B. (2021). Correlation of the mechanical and biological response in light-cured RBCs to receiving a range of radiant exposures: Effect of violet light. J. Dent..

[56] Par M., Repusic I., Skenderovic H., Tarle Z. (2019). Wavelength-dependent light transmittance in resin composites: Practical implications for curing units with different emission spectra. Clin. Oral. Investig..

[57] Ilie N. (2017). Impact of light transmittance mode on polymerisation kinetics in bulk-fill resin-based composites. J. Dent..

[58] Palin W.M., Leprince J.G., Hadis M.A. (2018). Shining a light on high volume photocurable materials. Dent. Mater..

[59] Emami N., Sjodahl M., Soderholm K.J. (2005). How filler properties, filler fraction, sample thickness and light source affect light attenuation in particulate filled resin composites. Dent. Mater..

[60] Musanje L., Darvell B.W. (2006). Curing-light attenuation in filled-resin restorative materials. Dent. Mater..

[61] Son S.A., Park J.K., Seo D.G., Ko C.C., Kwon Y.H. (2017). How light attenuation and filler content affect the microhardness and polymerization shrinkage and translucency of bulk-fill composites?. Clin. Oral. Investig..

[62] dos Santos G.B., Alto R.V., Filho H.R., da Silva E.M., Fellows C.E. (2008). Light transmission on dental resin composites. Dent. Mater..

[63] Harlow J.E., Rueggeberg F.A., Labrie D., Sullivan B., Price R.B. (2016). Transmission of violet and blue light through conventional (layered) and bulk cured resin-based composites. J. Dent..

[64] Rocha M.G., Roulet J.F., Sinhoreti M.A.C., Correr A.B., Oliveira D. (2021). Light Transmittance and Depth of Cure of a Bulk Fill Composite Based on the Exposure Reciprocity Law. Braz. Dent. J..

[65] Marovic D., Danicic P., Bojo G., Par M., Tarle Z. (2024). Monowave vs. Polywave Light—Curing Units: Effect on Light Transmission of Composite without Alternative Photoinitiators. Acta Stomatol. Croat..

[66] Par M., Repusic I., Skenderovic H., Milat O., Spajic J., Tarle Z. (2019). The effects of extended curing time and radiant energy on microhardness and temperature rise of conventional and bulk-fill resin composites. Clin. Oral. Investig..

[67] Marovic D., Bota M., Tarle F., Par M., Haugen H.J., Zheng K., Pavic D., Milos M., Cizmek L., Babic S. (2024). The influence of copper-doped mesoporous bioactive nanospheres on the temperature rise during polymerization, polymer cross-linking density, monomer release and embryotoxicity of dental composites. Dent. Mater..

[68] Plant C.G., Jones D.W., Darvell B.W. (1974). The heat evolved and temperatures attained during setting of restorative materials. Br. Dent. J..

[69] Hori M., Fujimoto K., Hori T., Sekine H., Ueno A., Kato A., Kawai T. (2020). Development of image analysis using Python: Relationship between matrix ratio of composite resin and curing temperature. Dent. Mater. J..

[70] Braga S., Oliveira L., Ribeiro M., Vilela A., da Silva G.R., Price R.B., Soares C.J. (2019). Effect of Simulated Pulpal Microcirculation on Temperature When Light Curing Bulk Fill Composites. Oper. Dent..

[71] Santos M., Fidalgo-Pereira R., Torres O., Carvalho O., Henriques B., Ozcan M., Souza J.C.M. (2024). The impact of inorganic fillers, organic content, and polymerization mode on the degree of conversion of monomers in resin-matrix cements for restorative dentistry: A scoping review. Clin. Oral. Investig..

[72] Leprince J.G., Palin W.M., Hadis M.A., Devaux J., Leloup G. (2013). Progress in dimethacrylate-based dental composite technology and curing efficiency. Dent. Mater..

[73] Thanoon H., Price R.B., Watts D.C. (2024). Thermography and conversion of fast-cure composite photocured with quad-wave and laser curing lights compared to a conventional curing light. Dent. Mater..

[74] Liu X., Sun Q., Zhang Y., Feng Y., Su X. (2023). Rapid RAFT Polymerization of Acrylamide with High Conversion. Molecules.

[75] Odum N.C., Ross J.T., Citrin N.S., Tantbirojn D., Versluis A. (2023). Fast Curing with High-power Curing Lights Affects Depth of Cure and Post-gel Shrinkage and Increases Temperature in Bulk-fill Composites. Oper. Dent..

[76] Ultradent (2021). Valo Cordless Instructions for Use.

[77] Par M., Prskalo K., Taubock T.T., Skenderovic H., Attin T., Tarle Z. (2021). Polymerization kinetics of experimental resin composites functionalized with conventional (45S5) and a customized low-sodium fluoride-containing bioactive glass. Sci. Rep..

[78] Par M., Gamulin O., Marovic D., Klaric E., Tarle Z. (2015). Raman spectroscopic assessment of degree of conversion of bulk-fill resin composites-changes at 24 hours post cure. Oper. Dent..

